# The Epidemiology of Sickle Cell Disease in Sub‐Saharan Africa: Current Knowledge and Gaps to be Filled

**DOI:** 10.1002/ajh.70212

**Published:** 2026-03-14

**Authors:** Brigitte Ranque, Leon Tshilolo, Thomas N. Williams

**Affiliations:** ^1^ Internal Medicine Department, Hôpital Européen Georges Pompidou, Assistance Publique Des Hôpitaux de Paris, Initiatives IdEx Globule Rouge D'Excellence (InIdex GR‐Ex) Université Paris Cité Paris France; ^2^ Institut de Recherche Biomédicale 1 Health, Kinshasa; Pediatrics Department Université Officielle de Mbujimayi Mbujimayi Democratic Republic of the Congo; ^3^ KEMRI‐Wellcome Trust Research Programme, Kilifi, Kenya Institute of Global Health Innovation, Imperial College London UK

**Keywords:** morbidity, mortality, outcome, sickle cell disease, SubSaharan Africa

## Abstract

Sickle Cell Disease (SCD) is highly prevalent in sub‐Saharan Africa. Epidemiological data remain sparse, but regional screening and research initiatives are expanding. Due to genetic, environmental, and socioeconomic factors, the disease course differs markedly from that in high‐income countries. Although mortality is improving and can be further lowered with simple interventions, it remains high, especially among undiagnosed children. Genetic factors, poor healthcare infrastructure, and poverty contribute to disease severity. While recent collaborative programs like SickleInAfrica offer hope, national policies that foster the training of healthcare workers, newborn screening, and access to treatment are crucial to reducing the burden of SCD across the region.

## Graphical abstract

What are the available data on incidence & prevalence prognosis risk factors of severity of sickle cell disease in sub‐Saharan Africa?

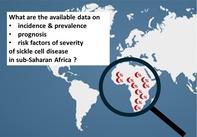

Globally, most patients with Sickle Cell Disease (SCD) are born in sub‐Saharan Africa (SSA). However, the incidence and types of SCD vary widely throughout this region and for multiple reasons, which include geographical, genetic, and socioeconomic factors; the course of SCD in SSA is markedly different from that in high income countries. In this review, we will focus on the prevalence and incidence of SCD in SSA, its clinical course, and the risk factors for its severity within the region. We also question the reliability of the available data.

## 1 Prevalence and Genotype Distribution of SCD in Sub‐Saharan Africa

Although SSA is home to most patients with SCD worldwide, the exact prevalence of the disease by region remains unknown. To date, although several pilot studies have been conducted in specific cities or regions (Table 1), and some countries are now on a path to universal screening [13, 16], no African country has yet implemented a comprehensive national screening program for SCD [16]. In Uganda, the US3 study demonstrated the feasibility of conducting a nationwide prevalence study for SCD based on > 320 000 residual dried blood spots collected from infants of HIV positive mothers [13], a concept that is now being expanded to all newborn children nationally, while similar initiatives have also been trialed in Tanzania and Malawi [18, 19]. Although largely focused on Anglophone countries, multinational newborn screening studies have recently been launched by the SickleInAfrica and CONSA consortiums [20], that should help gather more accurate SCD prevalence and incidence data within the region [20]. Therefore, while current estimates of the prevalence and incidence of SCD by region in SSA rely on relatively sparce and often biased data, more recent developments should soon lead to significant improvements.

**TABLE 1:** Sub‐Saharan newborn screening programs published in the past 10 years (PubMed search).

Country	City/region	1st author (citation)	Screening period	Number of screened babies	Prevalence of SCD
West Africa					
Gambia	7 regions and Banjul	Adegoke [1]	Apr 2023‐ Aug 2023.	1168	15 SS (1.3%) 2 SC (0.2%)
Liberia	Monrovia	Tubman [2]	NA Published in 2016	2785	33 SCD (1.2%) including 28 SS (1.0%) 2 SC (0.01%) 2 S*β* + (0.01%)
Ghana	Korle Bu Teaching Hospital	Segbefia [3]	Jul 2017‐Jul 2018	4427	79 (1.8%) presumptive SCD
Mali	Bamako	Guindo [4]	April–August 2021	3648	0.63% SS, 0.85% SC, 0.16% HbS/*β*+
Burkina Faso	Ouagadougou Bobo Dioulasso	Sawadogo [5]	2015–2019	11 337	215 SCD (1.9%) 60 SS (0.5%) 155 SC (1.4%)
Nigeria	Lagos	Oluwole [6]	Aug 2019‐Jan 2020	291	2 (0.8%) SCD including 1 SS (0.4%) 1 SC (0.4%)
Nigeria	Gwagwalada Area Council, Abuja	Nnodu [7]	July 2017‐Sept 2019	3603	51 (1%) HbSS, 4(< 1%) HbSC, 740 (21%) HbAS, 34 (1%) AC
Central Africa					
Angola	Luanda	Olaniyan [8]	NA Published in 2023	2000	34 SS (1.7%) 0 SC
Angola	Luanda	McGann [9]	Jul 2011‐Jun 2013	36 453	1.5% SS, 0.02% SC
RDC	Kisangani	Kasai [10]	Mar‐Apr 2022	1432	31 (2.2%) SS
RDC	Butembo and Beni	Mumbere [11]	Apr 2021‐Jan 2022	1195	2 (0.2%) SS
East Africa					
Tanzania	Bougando and Sekou Toure Mwanza (Northwest)	Ambrose [12]	Aug‐Sept 2014	919	13 (1.4%) SS 0 SC
Tanzania	Northwest	Ambrose [13]	Feb 2017‐May 2018	17 200	1.2% SS 0 SC 0 S/*β*+
Tanzania	Dar es Salaam	Nkya [14, 14]	Jul 2015‐Nov 2016	3981	31 (0.8%) SCD,
Tanzania	Dar es Salaam	Christopher [15]	NA Published in 2022	676	3 (0.4%) SS
Uganda	National	Ndeezi [16]	Febr 2014 – Mar 2015	97 631 including 66 670 before the age of 6 months	0.8% SS (before 6 months)
Uganda	National	Hernandez [17]	Feb 2014‐ Mar 2019	324 356 children aged 0–24 months	2036 (0.9%) SS 0 SC 0 S/*β*+
Malawi	Central Malawi	Tegha [18]	May‐Dec 2018	10 529 < 24 months	SS 14 (0.1%) AS 7.0% (range 3.9%–9.7% by district)

*Note*: Publications were found through a search in PubMed database, restricted to the past 10 years, using the following key words request: “[new‐born screening OR neonatal screening] and Africa, [sickle cell disease OR sickle cell anemia]” as well as citation lists in the retrieved publications.

Using data on the prevalence of SCD carriers (HbAS) in populations from the available literature, coupled with geostatistical modeling methods, Piel and colleagues estimated the annual number of SCA births in SSA at ~230 000 in 2010, corresponding to ~75% of all SCA births worldwide [21]. The same authors predicted that these numbers would rise significantly by 2050, due to population growth and increased survival [22]. More recently, again based on very partial data, the Global Burden of Disease Study presented updated prevalence and incidence estimates, suggesting that ~405 000 (95% CI 343000–478 000) babies were born with SCA in SSA in 2021, a 27.2% increase since 2000. The authors largely attributed this increase to population growth, rather than changes in age structure or disease frequency. Equatorial Guinea, Benin, Burkina Faso, Nigeria, Sierra Leone, and Togo all had estimated birth prevalences of > 2%, together totaling 44% of the global prevalence at birth. Countries with a prevalence of 1%–2% of live births included the Democratic Republic of the Congo (DRC) and Sao Tome and Principe in Central Africa, Ghana, Guinea, Angola, and Niger in West Africa, and Kenya in East Africa. Although these estimates were very uncertain because of the lack of reliable mortality data, they estimated that 5.68 million (95 CI 4.78–6.62) people were living with SCA in SSA in 2021, a 67.4% increase since 2000 [23]. Given recent improvements in life expectancy among children born with SCD, these are probably underestimates.

Useful as they are, these global prevalence data conceal considerable heterogeneity within countries ([10] and (Table 1)). For example, the Ugandan US3 study showed that the prevalence of SCA varied from 0.2% in the South to 1.5% in the East Central region [13]. Similarly, two newborn screening programs conducted in the DRC found a prevalence of 0.2% in Butembo and Beni compared to 2.2% in Kisangani [11, 24]. Finally, in Tanzania, the prevalence of SCD in newborns was 1.4% in Mwanza but only 0.8% in Dar es Salaam and even varied from 0.5% to 1.5% within the Northwest regions [18]. This variation might be explained by varying malaria selection pressure due to geographical characteristics such as altitude or hygrometry, and the appearance of the SCD mutation in different places of the continent at different times followed by genetic isolation due to endogamy, although none of those hypotheses have been proven.

The distribution of the different types of SCD also shows marked heterogeneity. For example, HbSC accounts for a significant proportion of SCD in many parts of West Africa [5] including Burkina Faso, where more than 70% of patients with SCD have the SC genotype [25], but is largely absent from other parts of the continent [5]. Moreover, though rarer, HbS/*β* ^0^ thalassemia and the milder form HbS/*β* ^+^ thalassemia account for a small proportion of SCD in specific parts of West Africa, including regions within Nigeria [26], Ghana [27], Liberia [28], Ivory Coast and Mali [29] HbS/*β* ^0^thalassemia has also been recently reported in Kenya, where it accounts for 10% of all SCD cases in the coastal region of Kilifi [30]. However, given that the phenotype of HbS/*β* ^0^thalassemia is so similar to that of HbSS disease [31], and that its diagnosis requires genetic typing that has not been widely undertaken in SSA, it is possible that the distribution of HbS/*β* ^0^thalassemia has been underestimated within SSA.

## 2 Mortality

The lifespan of patients with SCD in high‐income countries has improved considerably over the last 40 years, where in some it has now reached 50 years [32, 33]. For multiple reasons, including the implementation of neonatal screening programs, universal vaccination, and more effective treatments such as hydroxyurea, blood transfusion therapy and more recently, bone marrow transplantation, childhood mortality among those born with SCD in such countries is now very low [33, 34, 35]. By contrast, despite a lack of direct evidence, it has long been believed that most children born with SCD in SSA have been dying, largely undiagnosed, during early childhood [36]. There are many reasons for the lack of high‐quality epidemiological data. First, the absence of systematic neonatal screening means that most children born with SCD in SSA are only diagnosed based on clinical suspicion, often in later childhood. Many therefore are not treated early and may die before receiving a diagnosis. Even where neonatal screening projects have provided reliable estimates, mortality data have largely remained unavailable due to a lack of systematic follow‐up. Most long‐term prospective studies, therefore, only describe survivor cohorts, often in specialized hospital settings. Consequently, they do not provide an accurate picture of the natural history of the SCD, particularly in rural areas.

The very high mortality of children with SCD in SSA was first suggested by data from a malaria study conducted in the Garki region of northern Nigeria more than 50 years ago (1970–74) [37]. By screening 534 newborns and 2742 individuals from the general population to determine the prevalence of hemoglobin variants and genotypes, the study found only one child with SCD in the 1–4 year age group (rather than the six expected individuals) and only one among children aged 5 years and older (instead of the expected 53), suggesting that 98% of the children born with SCD had died before the age of 5 years. More recently, Grosse and colleagues reviewed data from ten studies conducted in SSA, including the Garki study, to compare the observed and expected frequencies of HbSS by age group, which they estimated from the observed proportion of HbAS individuals and the expected frequencies of HbSS based on Hardy Weinberg equilibrium [38]. They concluded that mortality before the age of five was between 50% and 90%. Among the studies included in this analysis, four were conducted after 2000, three of which were in rural areas where the estimated mortality remained very high. However, due to the very low frequency of HbSS overall, these surveys were not sufficiently powered to allow for precise mortality estimates.

More recently, in one of the few birth cohort studies of children with SCD reported from SSA, Uyoga and colleagues recruited almost 16 000 children from Kilifi County in Kenya who they followed passively until their 5th birthday. This cohort included 128 children with SCD in which group the mortality rate was 5.8 (95% CI 4.0–8.8) deaths per 100 person years of observation (PYO). In total, 29% of the children born with SCD died before reaching the age of 5 years [30]. Of note, approximately half of these children consented to being enrolled into a specialist SCD clinic where they were offered folic acid supplementation, pneumococcal prophylaxis with penicillin V and malaria prophylaxis with proguanil, while the other half declined to attend. Mortality was significantly lower in those who attended the clinic (2.9/100 PYO) than in those who did not (10.4/100 PYO) (Incidence Rate Ratio 0.26, 95% CI 0.11–0.62), suggesting that mortality can be substantially reduced with the introduction of just a few simple and affordable interventions.

**FIGURE 1:**
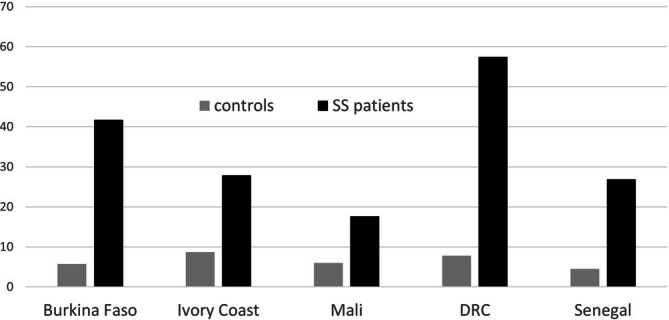
Estimated mortality rates before age 5 years (in %) by country among children with SCA and controls in the MIDAS Study. (adapted from Ranque et al., Lancet Haematol. 2022 [39]).

The MIDAS study proposed an alternative, indirect approach to estimating mortality among children with SCD within major cities in Burkina Faso, the DRC, Ivory Coast, Mali, and Senegal. Healthy women with at least three children that included at least one child with SCD were recruited. A similar group of women from the same neighborhood, who had the HbAA phenotype as established by point‐of‐care test, and who therefore had no children with SCD, were recruited as controls. Information about vital status was obtained for their 12 233 children. Following Mendel's law, it was assumed that in families with homozygous SCD children but healthy parents, on average 25% of live‐born children would be born with SCD. Based on this assumption, the under‐5 year and under‐10 year mortality rates in children with SCD were estimated at 36.4% [95% CI: 33.4–39.4] and 41.6% [31.5–51.7], respectively [39]. The relative risks of mortality among children with SCD were 5.4 and 5.8 respectively, with significant disparities between countries (Figure 1).

Another recent study used mathematical modeling to estimate mortality among children born with SCD in Nigeria, using data from a random selection of households from the national Demographic and Health Survey (DHS), through which 10 195 children were tested for SCD shortly after birth. Many assumptions were made to estimate the genetic link between participants and the Hb genotypes of 15 227 untested siblings, leading to wide confidence intervals. However, in broad agreement with the results from the MIDAS study, these authors estimated under‐5 years mortality among children with SCD at 49% (95% CI: 24–70) [40]. Finally, two recent studies have reported much lower rates of mortality in children with SCD who were followed in expert centers. Under‐5 mortality was estimated at 7.3 per 100 PYO (2.9% total deaths before the age of 5 years) in a study conducted in a major hospital with a SCD expert center in Dar es Salaam, Tanzania in 2010 [40], and at 3.5 per 100 PYO in children with a median age of 6 years in Dakar, Senegal in 2020 [41, 42]. Similarly, in the REACH trial investigating the use of hydroxyurea therapy in children with SCD < 10 years old from the DRC, Angola, Kenya and Uganda, mortality was 3.6 per 100 PYO in the pre‐intervention phase of the trial [43]. However, these three studies suffer from a major bias, as only diagnosed children who were followed in the hospital were included, meaning that children who died before being diagnosed were not included.

Overall, the survival of children with SCD in SSA has improved significantly in recent years, particularly in cities where dedicated care programs operate in pediatric centers. These results illustrate significant improvements in the quality of care provided to children with SCD in hospitals in SSA but also show that mortality remains substantially higher than it is in high‐income countries where, for example, more than 90% of children with SCD now reach adulthood in Europe [44, 45] and North America [46, 47]. This difference is probably due to the use of specific treatments such as hydroxyurea, blood transfusion, and, less often, bone marrow transplantation in high‐income countries, treatments that are much less readily available in SSA. Of note, bone marrow transplantation seems currently impossible to implement in SSA due to its huge cost and the high level of medical, biological, and pharmacological technicality required for conditioning, engraftment, and GVH monitoring, opportunistic infections detection, and treatment. Therefore, the recent reduction in mortality in SSA is probably attributable to general interventions such as parental education and vaccinations, as discussed below.

In high‐income countries, the causes of death related to SCD differ significantly between children and adults. Historically, infant mortality was largely due to infections, acute anemia, and strokes [35]. Because of the widespread use of penicillin or amoxicillin prophylaxis during the first 5 years of life and the introduction of vaccine programs for *Streptococcus pneumoniae* and *Haemophilus influenzae* , death from bacterial infections is now uncommon. Among adults, mortality is primarily due to acute chest syndrome (ACS) or chronic complications that include pulmonary hypertension, kidney failure, or liver disease [34, 35, 44, 46].

In SSA, studies describing the causes of death among individuals with SCD have largely been hospital based [48, 49]. There are no specific death registries for SCD in most SSA countries, so the proportion of SCD in the population's mortality by age group cannot be directly assessed as it has been in Europe and the United States during the last 30 years. Moreover, most patients die at home without seeing a doctor or a health care worker, so the causes of death are largely unknown.

Infectious diseases are the main cause of infant mortality in SSA, both in subjects with SCD and in the general population. In a pediatric admission study conducted in Dar es Salaam, Tanzania, the main causes of death in children with SCD were infections (primarily malaria) and acute anemia [41, 50]. Unlike carriers (HbAS), who have significant protection against severe malaria, children with SCD are at a higher risk of dying from malaria [51, 52, 53]. Therefore, malaria chemoprophylaxis is widely recommended for children with SCD in SSA, although the drugs vary from region to region. A recent randomized trial in Uganda and Malawi compared chemoprophylaxis with weekly dihydroartemisinin–piperaquine (DHA‐PQP) to monthly sulfadoxine–pyrimethamine (SP) in 725 children with SCD younger than 5 years. The incidence of clinical malaria was considerably lower in the DHA‐PQP group (8.8 versus 43.7 events per 100 person‐years), although the death rate (2%) was similar between the two groups [54]. In particular, participants in the DHA‐PQP group had more frequent lower respiratory tract infections than the SP group, probably because of the antibacterial effect of SP. Indeed, bacterial infections are also responsible for many deaths in children with SCD. In a large study documenting bacteremia in children with SCD in Kenya between 1998 and 2008, the most frequently isolated organisms were *S. pneumoniae* (41%), non‐typhoid salmonella species (18%), and *H. influenzae* type b (12%). Without penicillin prophylaxis, 23% of children with SCD died [55]. Another large study confirmed that *S. pneumoniae* and non‐typhoid salmonella species were the most common causes of death from bacterial infection in children with SCD in Nigeria, followed by enterobacter and acinetobacter species, and *Staphylococcus aureus* [56]. However, more recent studies of bacteremia conducted in children with SCD in The Gambia [57] and in Cameroon [58], found very few infections caused by either *S. pneumoniae* or *H. influenzae* , probably due to the recent introductions of vaccines directed against both of these pathogens.

In adults, a retrospective hospital‐based study conducted in Dakar between 2011 and 2020 found that the main causes of death (at an average age of 26 years) were acute anemia, followed by infections, ACS, and chronic kidney failure [41]. Acute anemia is often fatal due to difficulty accessing blood transfusions and to frequent infectious complications or allo‐immunization. Mortality from stroke is likely underestimated due to difficulties in accessing brain imaging and transcranial Doppler (TCD) velocity investigations [59].

## 3 Morbidity

Estimates of the prevalence and risk factors for the acute and chronic complications of SCD are also uncertain in SSA, having often been derived from cross‐sectional or retrospective studies in routine care settings which suffer from several biases.

### 3.1 Acute Vaso‐Occlusive Complications

The incidence of vaso‐occlusive crises (VOCs) has not been comprehensively described in SSA. In the CADRE study, which included more than 3000 SCD patients with a median age of 20 years in Cameroon, Côte d'Ivoire, Mali, Gabon and DRC [29, 60, 61, 62, 63], the mean number of VOCs (defined as pain lasting more than 48 h) was 1.8 (SD 2.4) episodes per year and the median was 1 [Q1: 0—Q3: 3]. VOCs were 30% more frequent in HbSS and HbS/*β* ^0^thalassemia patients compared to other forms of SCD. The prevalence of at least one lifetime episode of ACS was 33% in HbSS and HbS/*β* ^0^thalassemia patients (median age 20 years old) versus 16% in HbS/*β* ^+^thalassemia and 13% in HbSC patients (median age 25 years old). Noteworthy, because of the difficulty of differentiating the two conditions, infectious pneumonia was grouped together with ACS in this study. For both outcomes, considerable heterogeneity was observed between regions, whereby the patients living in Central Africa (Cameroon or DRC) had ORs of 4.1 [95% CI 2.3–7.4] for ACS and 1.9 [1.4–2.7] for the annual frequency of VOC after adjustment for hemoglobin phenotype, age, sex and hydroxyurea treatment (which involved less than 2% of this cohort) (Table 2 and Ranque B, personal communication). In the REACH trial [43], the incidence of VOC crises was 9.8 episodes/100 PYO among approximately 600 children < 10 years during the pre‐intervention period, while in the Kenyan birth cohort study [30], the incidence was 5.8 episodes/100 PYO among 128 children < 5 years.

**TABLE 2:** Main complications of sickle cell disease in four SSA countries (*Source:* Unpublished data from the CADRE Study).

Country	DRC *n* = 495	Cameroun *n* = 516		Mali *n* = 699		Senegal *n* = 719		Ivory coast *n* = 648	
SCD phenotype	SS‐S*β* ^0^	SC‐S*β* ^+^	SS‐S*β* ^0^	SC‐S*β* ^+^	SS‐S*β* ^0^	SC‐S*β* ^+^	SS‐S*β* ^0^	SC‐S*β* ^+^	SS‐S*β* ^0^
Sample size	493–0	2–11	503–0	256–39	377–27	44–7	662–6	209–102	234–103
Age (median [IQR], years)	14 [9–18]	12 [6–21]	13 [7–21]	21 [14–29]	17 [11–24]	13 [10–19]	15 [10–22]	22 [14–32]	15 [10–23]
Female (*n*, %)	251/493 (51)	7/13 (54)	251/503 (50)	152/295 (52)	229/404 (57)	24/51 (47)	359/668 (54)	186/311 (60)	188/337 (56)
BMI (median [IQR] kg/m^2^)	16.0 [15–28]	21.3 [19–23]	20.5 [19–22]	20.7 [19–24]	18.6 [17–21]	20.5 [18–24]	17.6 [16–19]	20.7 [18–24]	18.5 [17–21]
Hemoglobin (median [IQR], g/dl)	7.0 [6.3–7.9]	9.0 [7.2–9.4]	7.6 [6.7–8.6]	11.4 [10.5–12.5]	8.5 [7.7–9.6]	10.9 [10.2–11.7]	8.3 [7.5–9.2]	11.3[10.4–12.2]	8.1 [7.2–9.1]
eGFR (median [IQR], ml/min)	NA	131 [113–184]	159 [135–214]	111 [92–128]	133 [120–57]	118 [97–133]	130 [114–50]	130 [115–50]	177 [143–64]
Microalbuminuria (*n*, %)	28/149 (18.8)	2/13 (15.4)	197/503 (39.2)	69/295 (23.4)	152/404 (37.6)	5/51 (9.8)	154/668 (23.1)	30/311 (9.7)	54/337 (16.0)
VOC > 48 h past year (mean, SD)	3.1 ± 3.4	0.5 ± 0.7	1.5 ± 2.6	2.3 ± 2.5	2.6 ± 2.4	1.0 ± 1.2	1.0 ± 1.7	1.0 ± 1.3	1.3 ± 1.8
Priapism, lifetime (*n*, %)	29/446 (6.5)	1/6 (16.7)	43/251 (17.1)	26/143 (18.2)	26/175 (14.9)	4/27 (14.8)	32/308 (10.4)	8/125 (6.4)	9/150 (6.0)
Leg ulcer, lifetime (*n*, %)	36/448 (8.0)	1/13 (7.7)	63/501 (12.6)	13/295 (4.4)	38/404 (9.4)	1/27 (2.0)	29/668 (4.3)	5/311 (1.6)	8/33 (2.4)
Osteonecrosis, lifetime (*n*, %)	48/378 (12.7)	3/13 (23.1)	61/503 (12.1)	34/295 (11.5)	45/404 (11.1)	4/51 (7.8)	39/668 (5.8)	25/311 (8.0)	15/33 (4.5)
PAH (TRV > 3 m/s, %)	5/101 (5.0)	0	5 (3.7)	4 (5.3)	9 (9.6)	0	6 (4.9)	NA	NA
LV hypertrophy (*n*, %)	93/105 (91.0)	2 (100.0)	115 (82.1)	58 (70.7)	90 (94.7)	5 (50.0)	107 (72.8)	NA	NA
Stroke, lifetime (*n*, %)	13/457 (2.8)	1/13 (7.7)	16/503 (3.2)	2/295 (0.7)	3/404 (0.7)	0/51 (0.0)	11/668 (1.7)	0/311 (0)	1/33 (0.3)

Abbreviations: IQR: interquartile range; crise vaso‐occlusive crisis; TRV: tricuspid regurgitant jet velocity; eGFR: estimated glomerular filtration rate; PAH: pulmonary hypertension defined by TRV > 3 m/s. Left ventricular hypertrophy defined by LV mass (Devereux‘s formula) > 115 g/m^2^ for men et > 95 g/m^2^ for women.

### 3.2 Infections

As above, acute infections including malaria, lower respiratory and urinary tract infections, bacterial meningitis, and septicemia are particularly common and severe in subjects living with SCD in Africa [58, 64, 65] Septic arthritis and acute or chronic bacterial osteomyelitis also appear to be more common than they are in high‐income countries, although there are no reliable prevalence data for Africa. The main bacteria found are *S. aureus* and non‐typhoid salmonellae [65, 66, 67]. Unfortunately, partly because it is difficult to distinguish them from vaso‐occlusive events, osteoarticular infections are often diagnosed late and can therefore cause serious functional sequelae [68, 69].

Simple interventions can greatly reduce the burden of infectious diseases in children with SCD. Unfortunately, although malaria chemoprophylaxis is frequently recommended for these children [54] it is still rarely implemented. Likewise, despite its proven clinical utility [55], penicillin prophylaxis for young children with SCD is not uniformly prescribed [60, 70]. Noteworthy pneumococcal conjugate vaccine that was previously poorly available has recently been introduced in most SSA countries by the Global Alliance for Vaccines and Immunization (GAVI) so that vaccination coverage is now improving [71, 72].

### 3.3 Organ Damage

In contrast to patients in high‐income countries, patients in SSA rarely benefit from systematic screening for organ complications. Indeed, most have to self‐fund their care, so that the focus in most countries is on emergency care rather than on the prevention of chronic complications. Most of the organ monitoring that is now recommended in high‐income countries would be difficult to adhere to anyway due to the lack of human (ophthalmologists, nephrologists, doctors and nurses trained in transcranial Doppler), diagnostic (genetic diagnostic capability, testing for microalbuminuria) and imaging resources (MRI machines to assess hepatic iron overload, early avascular osteonecrosis or cerebral vasculopathy). As a result, the prevalence of most subclinical complications (such as retinopathy, aseptic osteonecrosis, cerebral vasculopathy, glomerulonephropathy, pulmonary hypertension, and ear, nose, and throat complications) is significantly underestimated in most of SSA. There is also imprecision on the nature of clinical complications (such as cerebral vasculopathy, cardiovascular and pulmonary disease). Furthermore, due to high SCD‐related mortality, there is significant survival bias. Finally, most studies that have described such complications have been conducted in expert centers where patients are better followed and are more often urban, better educated, and wealthier than the average population.

With these reservations in mind, the prevalence of the main chronic complications of SCD observed in the CADRE study are presented by country in Table 2. Overall, significant differences have been observed in disease severity not only between SCD phenotypes but also among the five countries, regardless of age and hemoglobin phenotype. For example, the prevalence of leg ulcers, a painful and debilitating skin complication, varied from 4.6% in Ivory Coast to 12% in Cameroon in the CADRE study, and 6.5% in another large cohort of SS children and adults in Nigeria [73]. These differences are likely explained by multiple factors, as discussed further below. In the CADRE study, the lifetime prevalence of clinically diagnosed stroke was very low (2%). Very low prevalences were also reported in recent retrospective analyses of large registries of patients with SCA: 1.4% in adults in Ghana (also clinically defined [74]), and 2.5% in children and adults in Nigeria, defined clinically and confirmed by brain imaging [73]. These studies probably underestimate the prevalence of stroke due to lack of systematic brain imaging and mortality bias. Prospective stroke data in children with SCD have been obtained from the REACH study, yielding much higher estimates—an incidence of 1.8 strokes / 100 PYO was observed before the introduction of hydroxyurea that reduced to 0.7/100 PYO within 3 years of hydroxyurea treatment. One consequence of cerebral vasculopathy is neurocognitive impairment. Studies in Uganda found a prevalence of 11% in SS children of mean age 5.5 years, which increased significantly with age, especially after 5 years [75, 76]. A much higher prevalence has been reported in adults. For instance, 38% of Cameroonian subjects with SCD aged 17–20 years had mild to severe cognitive dysfunction [77]. Although neurocognitive tests are poorly adapted to low income settings, and mild dysfunction may be attributed to other factors such as anxiety or depression, cognitive impairment has been consistently associated with high transcranial doppler velocity in African studies [75, 77, 78], suggesting that the prevalence of cerebral vasculopathy is significantly underestimated in available cohort studies.

## 4 Risk Factors for SCD Severity in SSA

Even in high‐income countries, our ability to predict the severity of SCD to guide management remains inadequate. The significant phenotypic variability observed between patients is likely due to a combination of genetic, environmental, and socio‐economic factors.

### 4.1 Translational Research Limitations in SSA

In total, more than 100 blood and urinary biomarkers have been correlated with at least one complication of SCD, but they often explain only a small portion of the overall variability of the disease [79] and are therefore not useful in clinical practice. Moreover, most are not feasible at scale in SSA. To be evaluated on a large scale, prognostic markers should preferably be accessible, inexpensive and reproducible, and should not require high‐level expertise. Nevertheless, much of the research in this area has tended to replicate studies conducted in high‐income countries, testing expensive biological markers, often under unreliable measurement conditions (such as prolonged transportation times due to poor road infrastructure), frequent power outages (which can interrupt the cold chain), non‐compliance with contamination precautions, unqualified personnel, and non‐repairable machines. Furthermore, some biomarkers may be less specific in the African context due to different environmental conditions, including the high infectious burden. For instance, a paradigm based on US data [80] links “hyper‐hemolysis” to vascular complications such as pulmonary hypertension and leg ulcers, and “hyper‐viscosity” to VOCs and osteonecrosis. This paradigm is not universally accepted and was challenged by the CADRE study, where such a dichotomy was not observed (after adjustment for Hb phenotype) when the level of hemolysis was determined using classical blood markers of hemolysis (LDH and bilirubin). The best indicator of organ damage was simply the level of anemia [60]. Likewise, in a hospital cohort from Dar es Salaam in Tanzania, the greatest risk factors for death were low hemoglobin (< 5 g/dL) and high total bilirubin (≥ 102 mmol/L) [41].

In 2017, the multinational SickleInAfrica consortium, based in Ghana, Nigeria, Tanzania, Mali, Uganda, Zimbabwe and Zambia, started to build large cohorts of patients living with SCD in SSA, and has now registered over 30 000 patients [81]. This registry includes demographic, clinical, and laboratory data. It will hopefully allow a better description of the natural history of SCD in those countries and will be well placed to test new biomarkers in the future. Likewise, ongoing treatment cohorts like REACH [43], NOHARM [82] and H‐PRIME [83] will yield valuable data on the incidence of long‐term complications and their risk factors in young patients treated with hydroxyurea.

### 4.2 Genetic Factors

The genetic prediction of SCD severity is still currently imperfect. Among the genetic factors that influence the variability in the clinical expression of SCD, those that influence hemoglobin and fetal hemoglobin levels are the best documented [84, 85, 86]. In particular, genetic persistence of fetal hemoglobin, which is associated with less severe hemolysis and anemia, is also linked to a lower frequency of VOCs and ACS [87, 88]. The co‐inheritance of α‐thalassemia also modulates the clinical severity of SCD by increasing the risks of VOC and osteonecrosis while lowering the risk of stroke, cerebrovascular disease, or glomerulonephropathy [1, 89, 90, 91]. Such genetic markers are distributed differently across regions in SSA, likely explaining some of the observed differences in severity. For example, the increased severity of SCD in the DRC or Cameroon that was observed in the CADRE and MIDAS studies could be partly related to the presence of the Bantu haplotype (CAR) in Central Africa, while SCD patients living in Mali, Senegal, and Côte d'Ivoire generally carry the Senegal (SEN) haplotype [2, 3]. The SEN haplotype includes the *HBG2* mutation predisposing to persistent fetal hemoglobin and is associated with a less severe phenotype. Indeed, high levels of fetal hemoglobin are more common in West Africa [4, 6]. By contrast, the prevalence of α‐globin deletion is lower in West Africa: in Mali and Senegal, one and two α‐globin deletions were detected in 19 to 26% and 2% of children, respectively [4, 7]. In the REACH trial, one and two α‐globin deletions were present in 48% and 11% of patients, respectively [8].

### 4.3 Socio‐Economic Factors

The MIDAS study found that the two main factors associated with lower infant mortality in sickle cell families were the family's economic level (evaluated by a global multidimensional poverty index) and the parents' level of education, particularly that of mothers [39]. In the CADRE study, hemoglobin level was also associated with parents' education level [63]. In Nigeria, the SPRING trial also showed that poverty was associated with more severe anemia [9]. Additionally, nutrient deficiencies associated with malnutrition and undernutrition were linked to disease severity and health‐related quality of life in adults and children with SCD [12]. Moreover, poor populations are exposed to prevalent infectious diseases (such as malaria, tuberculosis, and HIV) to a greater extent. Finally, absenteeism from school or work for patients and their caregivers can have a significant impact on their education or professional advancement. This results in a significant loss of income for the family, further complicating the management of SCD, as in SSA, the costs of healthcare are largely borne by patients or their families [14].

Heath systems also have a major impact on the course of SCD in SSA. Although quality care is available in centers of excellence in large cities, most people living with SCD in SSA do not have access to basic care for the condition. Some challenges are not specific to SCD such as costly healthcare and the low density of hospitals, doctors, imaging, and biological diagnostic facilities (Table 3), however, others have specific impacts on subjects with SCD. For instance, the low availability and high cost of blood transfusion, along with poor transfusion safety in terms of both immunological compatibility (no extensive typing of red blood cells) and the transmission of blood‐borne infections (inadequate screening for HIV, hepatitis B, and C), have a disproportionate impact on subjects with SCD, who are major users of transfusion facilities. Moreover, although hydroxyurea has been shown to be safe and effective in SSA [43] and is now becoming increasingly available, it remains widely underutilized in much of the region due to factors such as its high relative cost (given the extreme poverty of most families and the need for lifelong use), the need for frequent laboratory monitoring, supply chain disruptions, a fear of infertility, and a lack of knowledge among health care personnel [15, 60].

**TABLE 3:** General information on the economy, health system, and healthcare organization in the five participating countries in the MIDAS Study.

	Burkina Faso	Ivory coast	Mali	RDC	Senegal
Population (million inhabitants)	20.9	26.4	20.3	89.6	16.7
Poverty rate* ^#^	41.4%	39.5%	41.9%	63.9%	46.7%
Under 5 years old mortality rate	5.6%	7.0%	7.5%	11%	5.6%
Number of hospital beds per 1000 individuals* ^##^	0.2	0.3	0.1	0.8	0.3
Number of expert centres	> = 3	> = 5	> = 2	> = 3	> = 7
Genetic counseling	in expert centers	in expert centers	in expert centers	in expert centers	in expert centers
Newborn screening	No, except for pilot programs	Only in a few maternity hospitals	No, except for pilot programs	No, except for pilot programs	No, except for pilot programs

* Source: Word Bank data.

** Poverty rate: percentage of people with income below the poverty line (Source Word Bank data).

^#^ Source: World Health Organization 2019.

^##^ For comparison: 2.5 in UK, 2.9 in the USA et 5.9 in France.

The implementation of early interventions for infection prevention and parental education could also save many lives of children with SCD at a relatively low cost [30], but both require newborn screening, which, as previously mentioned, is not widely available. Nevertheless, even in the absence of newborn screening, children with SCD have significantly reduced mortality once diagnosed and managed in specific centers, thanks to vaccinations, the use of mosquito nets, antibacterial (penicillin) and antimalarial prophylaxis and parental education [30]. Unfortunately, even today, outside specific centers, many health care workers, including doctors, lack sufficient knowledge about SCD. A substantial effort is therefore needed to emphasize the importance of SCD during the initial professional education of doctors and nurses (see The Lancet Commission, section 5: Global strategies for training and educating health‐care providers: improving evidence‐based medicine and nursing care) [92].

### 4.4 Impact of the Environment

Many environmental factors can potentially influence the natural history of SCD, including meteorological variables (temperature, humidity, wind speed, or precipitation), air quality, altitude, and the prevalence of infectious diseases. However, the evidence supporting these effects is weak [17]. Most of the data generated so far on this subject comes from Europe and the United States, although studies are beginning to emerge from other countries with a high prevalence of SCD [93].

In SSA, the tropical climate is by itself potentially harmful for patients with SCD, in particular because of the dehydration risk and high infectious burden. This issue is getting worse, with global warming being responsible for more frequent and longer droughts that exacerbate economic inequalities. However, although based on sickle cell pathophysiology and expert recommendations only it is often considered important to avoid dehydration as a precipitating cause of VOC, there is currently no evidence that people with SCD living in dry regions have worse outcomes than others.

Pollution is becoming a major issue as well. Waste management is often very poor and plastic garbage accumulates or is burned, causing toxic fumes. Road congestion is common due to poor infrastructure and aging vehicles with high carbon and microparticle emissions. These issues are particularly visible in cities, which represent specific ecosystems where populations can potentially benefit from easier access to health infrastructure but also suffer from poor air quality and drastic socio‐economic inequalities [94]. The impact of climate change and pollution on SCD is currently unknown in SSA but certainly deserves specific studies.

## 5 Conclusion

For various reasons, including environmental, genetic, and socio‐economic factors, the clinical course of SCD is very different in SSA to that in high‐income countries. Despite fragmented and often biased epidemiological data, there are strong arguments to suggest that the morbidity and mortality associated with SCD are significant in SSA.

For a long time, SCD has been widely neglected by science funding agencies, and few funds have been available for studies in SSA to identify risk factors that could potentially guide patient follow‐up and validate cost‐effective interventions to optimize the scarce healthcare resources in these countries. In the meantime, in the absence of locally generated data, care recommendations have largely been adapted from those used in high‐income countries, even though some may not be appropriate in the African context. However, SCD‐specialists from different countries in SSA have come together to foster research and to improve care. For example, the REDAC network was established more than 10 years ago to promote research, health strategies and collaborations between hospitals, research centers, advocacy groups, and industry partners in Central Africa. This network has the advantage of uniting both French‐ and English‐speaking partners where co‐operation has been historically limited because of language barriers. More recently, a large research consortium known as SickleInAfrica has also been launched, supported by significant funding from the National Heart, Lung, and Blood Institute in the USA. This consortium holds great promise in terms of research and care improvements that will be tailored to SSA. It is a good example of international support based on skill transfer and not solely on ad hoc health interventions, as it has too often been the case until now.

Unfortunately, a large part of the poor prognosis of SCD in SSA countries is related to poor socio‐economic conditions and the weaknesses of the healthcare systems. Training healthcare workers, implementing systematic neonatal screening, providing low‐cost hydroxyurea, and increasing the availability and safety of blood transfusions are all necessary steps to improving the situation of patients [92]. As noted in the WHO report on the African region sickle‐cell strategy from 2010 to 2020, while all 23 countries defined as high burden (an HbAS prevalence of between 20% and 30%) had established a SCD unit in their Ministries of Health, only eight countries had allocated annual budget funds for health promotion of SCD, and only five had allocated funding for newborn screening (WHO regional office for Africa, AFR/RC70/INF.DOC/3, 2020). It is therefore necessary for all governments in SSA to consider this public health problem and implement the necessary health policies at the national level.

## Funding

TNW is funded through a Senior Fellowship (202 800/Z/16/Z) from the Wellcome Trust.

## Disclosure

Permission to Reproduce Material From Other Sources: Table 2 and Figure 1 are adapted from personal data, Table 3 is in Supplementary Inforamation of ref. [60].

## Ethics Statement

The authors have nothing to report.

## Consent

The authors have nothing to report.

## Conflicts of Interest

The authors declare no conflicts of interest.

## Data Availability

The authors have nothing to report.
